# Characterization of the Sterol and Phosphatidylinositol 4-Phosphate Binding Properties of Golgi-Associated OSBP-Related Protein 9 (ORP9)

**DOI:** 10.1371/journal.pone.0108368

**Published:** 2014-09-25

**Authors:** Xinwei Liu, Neale D. Ridgway

**Affiliations:** Departments of Pediatrics, Biochemistry and Molecular Biology, Dalhousie University, Halifax, Nova Scotia, Canada; University of Geneva, Switzerland

## Abstract

Oxysterol binding protein (OSBP) and OSBP-related proteins (ORPS) have a conserved lipid-binding fold that accommodates cholesterol, oxysterols and/or phospholipids. The diversity of OSBP/ORPs and their potential ligands has complicated the analysis of transfer and signalling properties of this mammalian gene family. In this study we explored the use of the fluorescent sterol cholestatrienol (CTL) to measure sterol binding by ORP9 and competition by other putative ligands. Relative to cholesterol, CTL and dehydroergosterol (DHE) were poor ligands for OSBP. In contrast, both long (ORP9L) and short (ORP9S) variants of ORP9 rapidly extracted CTL, and to a lesser extent DHE, from liposomes. ORP9L and ORP9S also extracted [^32^P]phosphatidylinositol 4-phosphate (PI-4P) from liposomes, which was inhibited by mutating two conserved histidine residues (HH_488,489_AA) at the entrance to the binding pocket but not by a mutation in the lid region that inhibited cholesterol binding. Results of direct binding and competition assays showed that phosphatidylserine was poorly extracted from liposomes by ORP9 compared to CTL and PI-4P. ORP9L and PI-4P did not co-localize in the trans-Golgi/TGN of HeLa cells, and siRNA silencing of ORP9L expression did not affect PI-4P distribution in the Golgi apparatus. However, transient overexpression of ORP9L or ORP9S in CHO cells, but not the corresponding PI-4P binding mutants, prevented immunostaining of Golgi-associated PI-4P. The apparent sequestration of Golgi PI-4P by ORP9S was identified as a possible mechanism for its growth inhibitory effects. These studies identify ORP9 as a dual sterol/PI-4P binding protein that could regulate PI-4P in the Golgi apparatus.

## Introduction

Lipids and sterols are exchanged between cellular organelles in transport vesicles or by soluble binding proteins that mediate monomeric transfer. Lipid and sterol binding/transport proteins are grouped into several large gene families that are structurally diverse with respect to their lipid binding folds and ancillary regulatory domains. Oxysterol binding protein (OSBP) and OSBP-related proteins (ORPs) comprise one such multigene family expressed in a variety of eukaryotic phyla [1], [2]. OSBP/ORPs are characterized by a lipid-binding fold consisting of a β-barrel with a hydrophobic interior that is capped with a flexible lid that effectively shields sterols and phospholipid acyl-chains from the aqueous environment of the cell [3], [4]. In addition to the lipid-binding domain, OSBP/ORPs also transiently interact with specific protein and lipid determinants on organelles. OSBP and most ORPs have a two-phenylalanine in an acid tract (FFAT) motif that binds vesicle-associated membrane protein-associated protein (VAP), a type II endoplasmic reticulum (ER)-associated protein [5], [6]. Members of the family also have N-terminal pleckstrin homology (PH) domains that bind phosphatidylinositol polyphosphates and ADP-ribosylation factor in the Golgi apparatus and other organelles [7], [8]. The domain organization of OSBP/ORPs indicates that their primary function is lipid transport and/or signalling that is mediated by sequential or simultaneous interaction with organelle membranes (reviewed in [9]).

OSBP and its mammalian and yeast homologues are known to bind oxysterols, cholesterol and ergosterol [10]–[13]. However the lack of direct contact between sterol hydroxyl groups and key residues in the hydrophobic β-barrel of Osh4 suggested that other lipids could be accommodated [3]. Recent structural and interactome mapping of yeast Osh proteins supports this concept. In addition to sterols, the acyl-chains of phosphatidylinositol 4-phosphate (PI-4P) extend into the hydrophobic β-barrel of Osh4, with the inositol 4-phosphate headgroup contacting a pair of histidine residues at the tunnel entrance [14]. The absolute conservation of this histidine pair in OSBP homologues across all phyla suggests a common PI-4P binding activity that could be involved in exchange of sterols and PI-4P between the Golgi apparatus and other organelles [14], [15]. The Osh3 β-barrel is too narrow to accommodate a sterol and exclusively binds PI-4P [4]. Similarly, Osh6 and Osh7 bind and transfer phosphatidylserine (PS) [16] but bind sterols weakly compared to other Osh proteins [11].

The mammalian OSBP/family is encoded by 12 genes, some of which have numerous splice and promoter variants that further contribute to the function diversity. OSBP, the founding member of the family, and its closest paralogue ORP4 are well-characterized high-affinity receptors of 25-hydroxycholesterol (25OH) and cholesterol [13], [17]. Similar to Osh4, OSBP also binds PI-4P competitively with cholesterol [18], and catalyzes the reciprocal transfer of PI-4P and dehydoergosterol (DHE) between liposomes *in vitro* [15]. In a cellular context, OSBP could catalyzes the exchange of cholesterol and PI-4P between the ER and Golgi apparatus, respectively, with Sac1 hydrolysis of PI-4P in the ER providing energy for the transfer of cholesterol to the Golgi apparatus against a concentration gradient [15]. OSBP regulation of Golgi cholesterol and sphingomyelin homeostasis involves sterol transport activity and recruitment of ceramide transfer protein to the organelle by activation of PI 4-kinase IIα activity [19]–[21]. ORP9L binds and transfers cholesterol *in vitro* and is localized to the trans-Golgi and trans-Golgi network (TGN) where it regulates protein secretion and the cholesterol content of post-Golgi compartments [22]. A truncated promoter variant termed ORP9S, which is missing the N-terminal PH domain and not expressed in the Golgi apparatus, is implicated in control of Akt signalling and cell proliferation [22], [23]. Unlike OSBP, ORP9L and ORP9S do not bind oxysterols *in vitro* nor are they involved in sphingomyelin regulation by oxysterols or cholesterol [22]. To understand the function of ORP9L and ORP9S, we investigated ligand binding properties using a Forster resonance energy transfer (FRET) assay with the fluorescent sterol cholestatrienol (CTL) and direct binding of radiolabelled phospholipids. Results show that ORP9L and ORP4S competitively extract sterols and PI-4P from liposomes, and can sequester or alter the metabolism of Golgi-associated PI-4P in cultured cells.

## Materials and Methods

### Materials

Egg yolk phosphatidylcholine (PC), porcine brain PS, egg yolk phosphatidylethanolamine (PE), 1,2-dioleoyl lactosyl-PE, 1,2-dioleoyl dansyl–PE, porcine brain PI-4P, cholesterol and DHE were purchased from Avanti Polar lipids (Alabaster, AL). CTL was prepared as previously described [24]. The anti-PI-4P mouse monoclonal antibody was purchased from Echelon Biosciences (Salt Lake City UT). [^3^H]Cholesterol and [^32^P]PO_4_ were purchased from Perkin-Elmer (Waltham, MA). ON-TARGETplus human OSBPL9 siRNAs were purchased from Dharmacon (Lafayette, CO). Odyssey blocking buffer and IRDye 680- and 800-conjugated secondary antibodies were obtained from LI-COR Biosciences (Lincoln, NE). Preparation of the rabbit anti-ORP9 antibody was previously described [25]. pENTR/D-ORP9L-HH_488,489_AA and pcDNA-ORP9L- HH_488,489_AA (ORP9L-HH/AA) were made by mutagenesis of the respective wild-type vectors (QuikChange II XL site-direct mutagenesis kit, Stratagene, La Jolla, CA) using forward and reverse primers (GCTGAGCAGGTTTCCGCTGCTCCACCCATTTCAGCC and GGCTGAAATGGGTGG AGCAGCGGAAACCTGCTCAGC, Integrated DNA Technologies, Coralville, IA) and verified by sequencing.

### Cell culture and recombinant protein expression

HeLa cells were cultured in DMEM containing 10% fetal bovine serum (FBS) at 37°C in a 5% CO_2_ atmosphere. ORP9L expression was silenced in HeLa cells by transfection using Trans-IT TKO transfection reagent (Mirus, Madison, WI) and a pool of three ORP9L siRNA duplexes (100 nM) or a non-targeting (NT) siRNA (100 nM) for 48 h as previously described [23]. Chinese hamster ovary (CHO) cells cultured in DMEM with 5% FCS and 34 µg/ml proline were transiently transfected with ORP9L expression vectors using Lipofectamine 2000. CHO cells expressing V5-tagged ORP9L and ORP9S under the control of the TET-on transactivator were cultured and induced with doxycycline as described [22].

Sf21 cells were cultured in SF900-II medium containing 5% (v/v) FBS, 10 µg/ml G418 and 0.25 µg/mL fungizone (Sf21 medium) in suspension at 27°C. pENTR/D-ORP9L-HH_488,489_AA was recombined with Baculodirect linear DNA with a C-terminal V5-His-tag and expressed in Sf21 cells to prepare baculovirus (Invitrogen, Burlington, ON). Baculovirus-encoded OSBP, ORP9L and ORP9S were expressed in Sf21 cells and purified by Talon affinity chromatography as previously described [22]. Hexahistidine-SUMO3-tagged Osh6 was expressed and purified from bacteria as previously described [16]. Protein purity was verified by SDS-8%PAGE and quantified using a modified Lowry assay [26].

### Sterol competition assays

Purified OSBP (20 pmol) was incubated for 2 h at 20°C with 100 nM of [^3^H]cholesterol and increasing amounts of unlabelled cholesterol, DHE or CTL in 10 mM HEPES (pH 7.4) and 300 mM KCl (binding buffer) containing 2% (w/v) PVA and 0.05% Triton-X 100. Each assay then received 25 µl of Talon metal affinity resin (1∶1, v/v) with constant mixing for 25 min. After brief centrifugation, supernatants were removed by aspiration and the Talon resin was washed 3 times with 300 µl of binding buffer at 4°C. OSBP bound to Talon resin was eluted with 100 µl of binding buffer containing 150 mM imidazole and OSBP-bound [^3^H]cholesterol in the supernatant was measured by liquid scintillation counting.

### CTL extraction from liposomes

Liposomes were prepared by combining phospholipids and CTL in a glass tube and drying under nitrogen. The lipid film was hydrated in liposome buffer (25 mM HEPES, pH 7.4, 150 mM NaCl, 1 mM EDTA) to a final concentration of 0.5 mM and vortexed every 5–10 min for 1 h at 20°C. Unless otherwise indicated liposomes were composed of PC/PE/PS/CTL/dansyl-PE (65∶20∶10∶2.5∶2.5, mol/mol) and prepared by extrusion through a 400 nm polycarbon membrane using the Lipofast system (Avestin, Ottawa ON). Liposomes were stored at 4°C for no more than 48 h and centrifuged at 15,000×*g* for 5 min prior to the start of experiments.

FRET assays were conducted using a Cary Eclipse fluorescence spectrophotometer equipped with a single-cell Peltier accessory to maintain temperature at 30°C and a detector setting of 900 V. The fluorescent cholesterol analog CTL (324/370 nm, excitation/emission) and dansyl-PE (370/520 nm, excitation/emission) were used as a FRET pair. Liposomes (0.05 mM) and proteins (0–2.5 µM) in a final volume of 60 µl of liposome buffer were combined in a micro quartz cuvette, excited at 324 nm (5 nm slit width) and emission at 520 nm (10 nm slit width) was measured at 20–30 sec intervals over 5 min. FRET emission was corrected by subtracting background (liposomes without CTL) and was expressed as a percent of maximal extraction by 1 mM methyl-β-cyclodextrin (CD). Extraction curves were fit using a one-phase exponential decay model (GraphPad Prism 5 Software).

### Liposome extraction assays using radiolabeled phosphatidylserine (PS) and PI-4P

[^32^P]-labelled PI-4P was isolated and purified from HeLa cells radiolabeled with [^32^P]PO_4_ for 16 h [18]. [^3^H]Serine-labeled PS was isolated from several 60 mM dishes of HeLa cells that were incubated with [^3^H]serine (40 µCi/ml) for 6 h. Total lipid extracts were prepared from cells, resolved by thin-layer chromatography in CHCl_3_/methanol/acetic acid/water (60∶40∶4∶1, v/v), and [^3^H]PS identified by migration with an authentic standard and extracted from silica gel scrapings with CHCl_3_/methanol, 2∶1, v/v). Liposomes composed of PC/PE/[^3^H]PS/lactosyl-PE (65∶20∶5∶10, mol/mol) or PC/PE/PS/lactosyl-PE/[^32^P]PI-4 (59.5∶20∶10∶10∶0.5, mol/mol) were made by extrusion as described above. The specific activity of [^3^H]PS and [^32^P]PI-4P in liposomes was 8 and 40 dmp/pmol, respectively. OSBP, ORPs or Osh6 (100 pmol) was incubated with 3 µg of fatty acid-free BSA and radiolabelled liposomes (0.1 mM) in liposome buffer at 25°C [18]. After 20 min, 10 µg of *R. communis* agglutinin was added on ice for 15 min and the liposomes were sedimented by centrifugation at 15,000×*g* for 5 min. [^32^P]PI-4P or [^3^H]PS was measured in the supernatant by scintillation counting and corrected for background binding in the absence of protein. [^3^H]PS extraction was also corrected for non-specific recovery of liposomes in the supernatant by inclusion of a [^14^C]PC tracer in the liposome preparation. Percent extraction was calculated based on total [^3^H]PS or [^32^P]PI-4P input into each assay.

### Immunoblotting

Cells were lysed in 0.8% SDS (w/v), 25 mM Tris-HCl (pH 6.8) 4% glycerol (v/v), 2% β-mercaptoethanol (v/v), heated at 95°C for 5 min, resolved by SDS-8%PAGE and transferred to nitrocellulose. Filters were incubated in Odyssey blocking agent diluted in TBS (20 mM Tris-HCl and 150 mM NaCl) (1∶5, v/v) and 0.01% Tween-20 containing ORP9 polyclonal and actin monoclonal antibodies. Goat anti-rabbit IRDye 680- and goat anti-mouse 800-conjugated secondary antibodies were used to detect ORP9 and actin using the Odyssey Infrared Imaging system (LI-COR Biosciences, Lincoln NE).

### Immunofluorescence microscopy

CHO or HeLa cells cultured on glass coverslips were fixed in PBS (10 mM sodium phosphate and 150 mM NaCl, pH 7.4) containing 2% formaldehyde (w/v) for 10 min and permeabilized and quenched in 2 ml of PBS containing 20 µg/ml digitonin and 100 mM glycine for 20 min at room temperature. Coverslips were blocked in PBS containing 1% BSA (w/v) for 1 h, and probed with an anti-PI-4P monoclonal antibody for 16 h at 4°C followed by a goat anti-mouse IgM Alexafluor594 secondary antibody for 1 h. Endogenous ORP9L or overexpressed ORP9 was detected with a polyclonal primary antibody and goat anti-rabbit Alexafluor488 secondary antibody. Coverslips were mounted using Mowiol 4–88. Images of HeLa cells were captured using a Zeiss Axiovert 200M inverted microscope equipped with a 63x oil emersion objective and Hamamatsu Orca R2 camera. Images of CHO cells were captured using a Zeiss LSM 510 Meta laser scanning confocal microscope (0.8 µm sections) equipped with 100x oil emersion objective. Fluorescent intensity of PI-4P immunostaining was quantified using Image J (v1.46). The ‘analyze particles’ command was used to quantify intensity of PI-4P staining of individual cells outlined in 16-bit images that were processed for background and threshold.

## Results

### Binding of fluorescent sterols by ORP9

**Figure 1:**
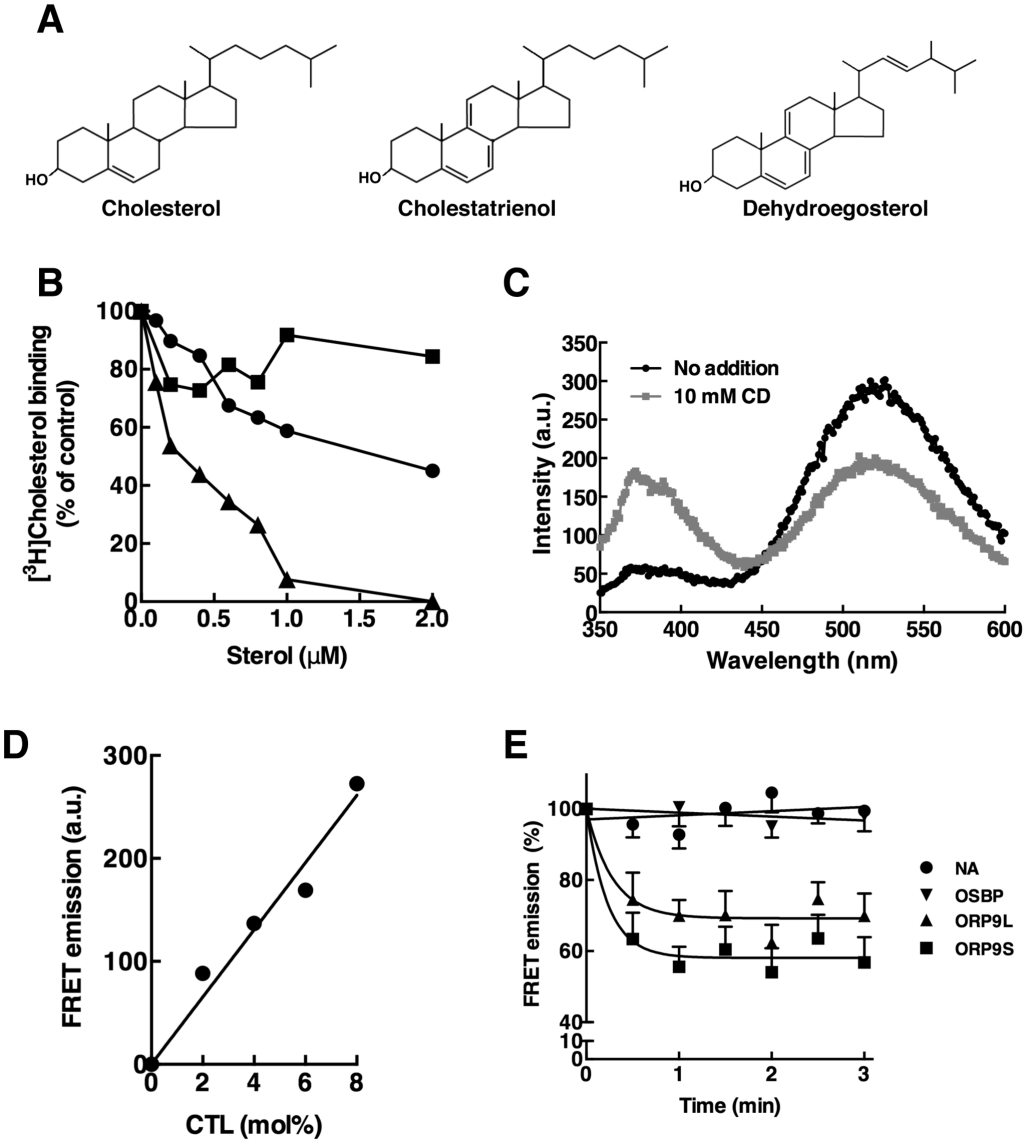
A FRET-based sterol extraction assay for ORP9. A, structure of cholesterol, DHE and CTL. B, OSBP (20 pmol) was incubated with 100 nM [^3^H]cholesterol and increasing concentrations of unlabeled CTL (l), DHE (▪) or cholesterol (▴). [^3^H]Cholesterol binding is expressed as a percentage of specific binding in the absence of unlabeled sterols that was corrected for non-specific binding in the presence of 40-fold excess of unlabeled cholesterol. C, liposomes containing 5 mol% CTL and 2.5 mol% dansyl-PE were incubated with no addition (black circles) or 10 mM CD (grey circles) and scanned from 350 to 600 nm. D, corrected FRET emission at 520 nm for liposomes (0.05 mM) containing increasing mol% CTL and 2.5 mol% dansyl-PE. E, liposomes (0.1 mM) containing 2.5 mol% CTL and 2.5 mol% dansyl-PE were incubated with no addition (NA), OSBP, ORP9L or ORP9S (2 µM) at 30°C. Corrected FRET emission is expressed as a percentage of the time 0 value. Results are the mean and SEM from three experiments and fit to linear (OSBP) or one-phase exponential decay (ORP9L and ORP9S).

Previous evidence of ORP9-mediated extraction and transfer of cholesterol relied on the measurement of soluble or liposome-associated [^3^H]cholesterol after sedimenting the donor or acceptor liposomes [22]. Isotope-based assays are fraught with technical problems, such as protein binding to and aggregation of liposomes that limits their usefulness for measuring sterol flux *in vitro*. As an alternative, the fluorescent sterol analogs CTL and DHE (Fig. 1A) were evaluated as ligands for OSBP and ORP9 using a liposome-based assay and the FRET acceptor dansyl-PE. CTL mimics the membrane behaviour of cholesterol [27] and is transferred between membranes by NPC2 [28]. DHE was used in liposomal extraction and transfer assays for ORP5, Osh4p and OSBP [11], [15], [29]. ORP9L and ORP9S do not bind cholesterol or 25OH that is dispersed in solution and thus a direct competition assay could not be used to test their relative affinity for fluorescent sterols [22]. As an alternative, we determined the relative affinity of OSBP for CTL and DHE using an assay in which binding of [^3^H]cholesterol (100 nM) was competed by increasing concentrations of unlabelled cholesterol, CTL or DHE dispersed in Triton X-100 (Fig. 1B). As expected a 20-fold excess of unlabelled cholesterol completely competed out [^3^H]cholesterol binding by OSBP. [^3^H]Cholesterol binding was inhibited by 50% in the presence of a 20-fold excess (2 µM) of CTL. Interestingly, 250 nM to 750 nM DHE inhibited [^3^H]cholesterol binding by only 10–15%. Based on these results, CTL was chosen as a FRET donor since it was a preferred ligand for OSBP and structurally similar to cholesterol.

**Figure 2:**
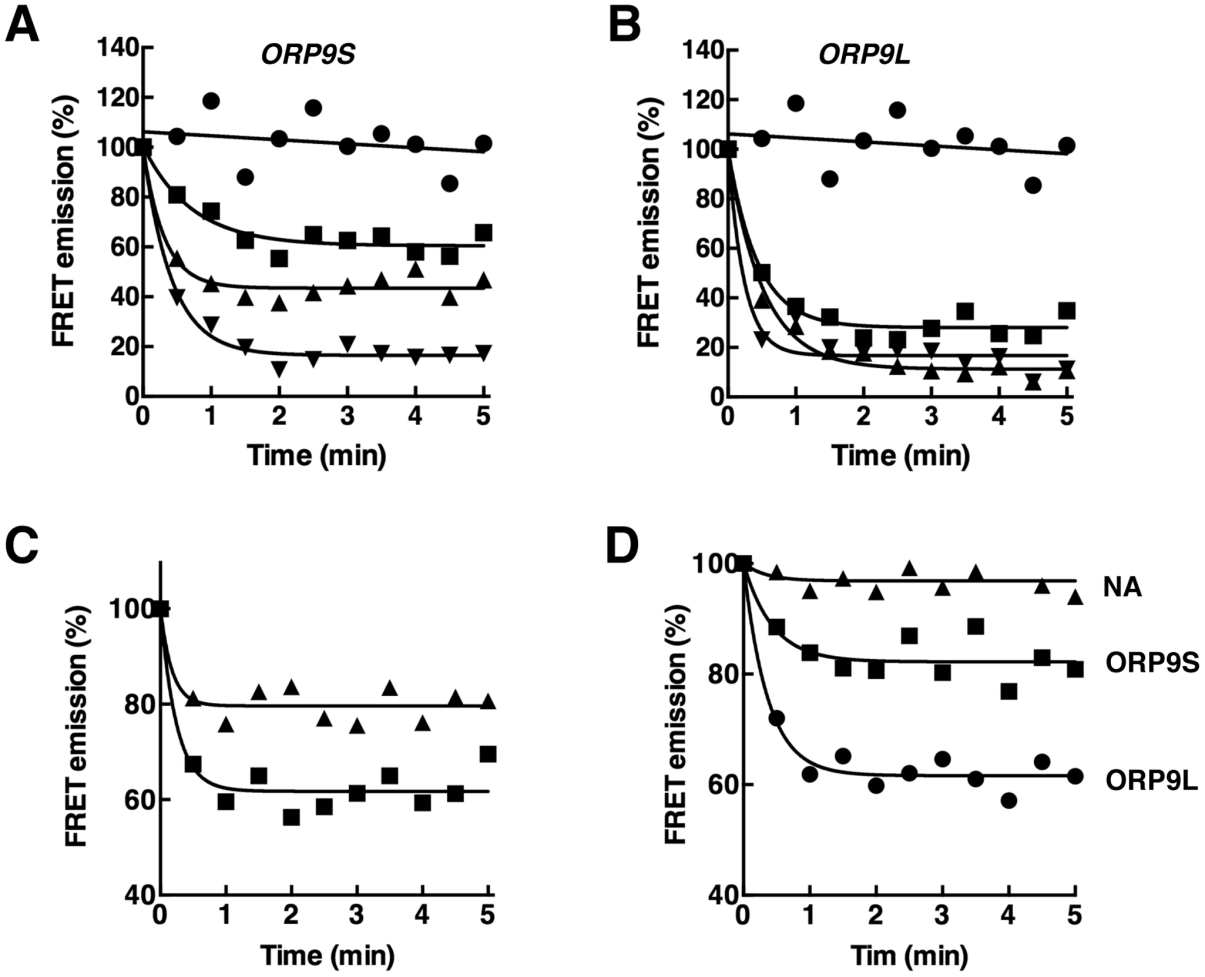
Dose–dependent extraction of CTL from liposomes by ORP9S and ORP9L. A and B, extraction of CTL from liposomes by 0 (•), 0.25 (▪), 0.5 (▴) or 1.0 µM (▾) ORP9S or ORP9L was quantified as described in the legend to Fig. 1E. Results are the mean of 3–5 experiments. C, ORP9L extraction of CTL from liposomes containing 0 (▪) or 2.5 mol% cholesterol (▴). Results are the mean of 3 experiments. D, extraction of 2.5 mol% DHE from liposomes by 0.5 µM ORP9L and ORP9S. Results are the mean 3 for experiments. For the purpose of clarity, error bars are not shown in panels A–D.

FRET between donor CTL and acceptor dansyl-PE was demonstrated with liposomes containing 5 mol% CTL and 2.5 mol% dansyl-PE treated without or with cyclodextrin (CD) (Fig. 1C). In the case of untreated liposomes (black line), excitation at 324 nm and scanning the 350–600 nm interval revealed a strong dansyl-PE emission at 520 nm due to energy transfer and a CTL emission at 370 nm. Addition of 10 mM of CD (grey line) reduced the FRET emission at 520 nm and increased the CTL emission at 370 nm indicating efficient extraction of CTL from liposomes. Extraction of CTL by 10 mM CD was used to establish the maximum and minimum FRET for the calculation of percent extraction by OSBP and ORP9. The relationship between FRET emission and CTL content of liposomes is shown in Fig. 1D. FRET and CTL content of liposomes were proportional indicating that changes in the 520 nm emission directly reflect the CTL content of liposomes. CTL extraction from liposomes containing 2.5 mol% CTL and 2.5 mol% dansyl-PE was initiated by addition of purified OSBP, ORP9L and ORP9S (Fig. 1E). In the absence of OSBP or ORP9, the baseline was stable indicating negligible photobleaching. OSBP did not decrease the FRET signal but both ORP9L and ORP9S extracted CTL based on a rapid 30–40% reduction in the 520 nm emission that was complete in 60–90 s. Increasing the OSBP concentration or altering the composition of liposomes failed to improve CTL extraction. Both ORP9 variants displayed robust extraction of CTL using the FRET assay (Fig. 2A and B). ORP9S extracted CTL in a dose-dependent manner to a maximum of 80% at 1 µM (Fig. 2A), while 0.25–1.0 µM ORP9L extracted a maximum of 70–90% (Fig. 2B). Compared to cholesterol-free liposomes, the inclusion of an equimolar amount of cholesterol (2.5 mol%) in liposomes caused a 50% inhibition of CTL extraction by ORP9L (Fig. 2C). ORP9L and ORP9S (0.5 µM) also extracted DHE from liposomes, although less efficiently than it did CTL (compare to 0.5 µM ORP9L and ORP9S in Fig. 2A and B) (Fig. 2D).

### ORP9 binds PI-4P competitively with CTL

**Figure 3:**
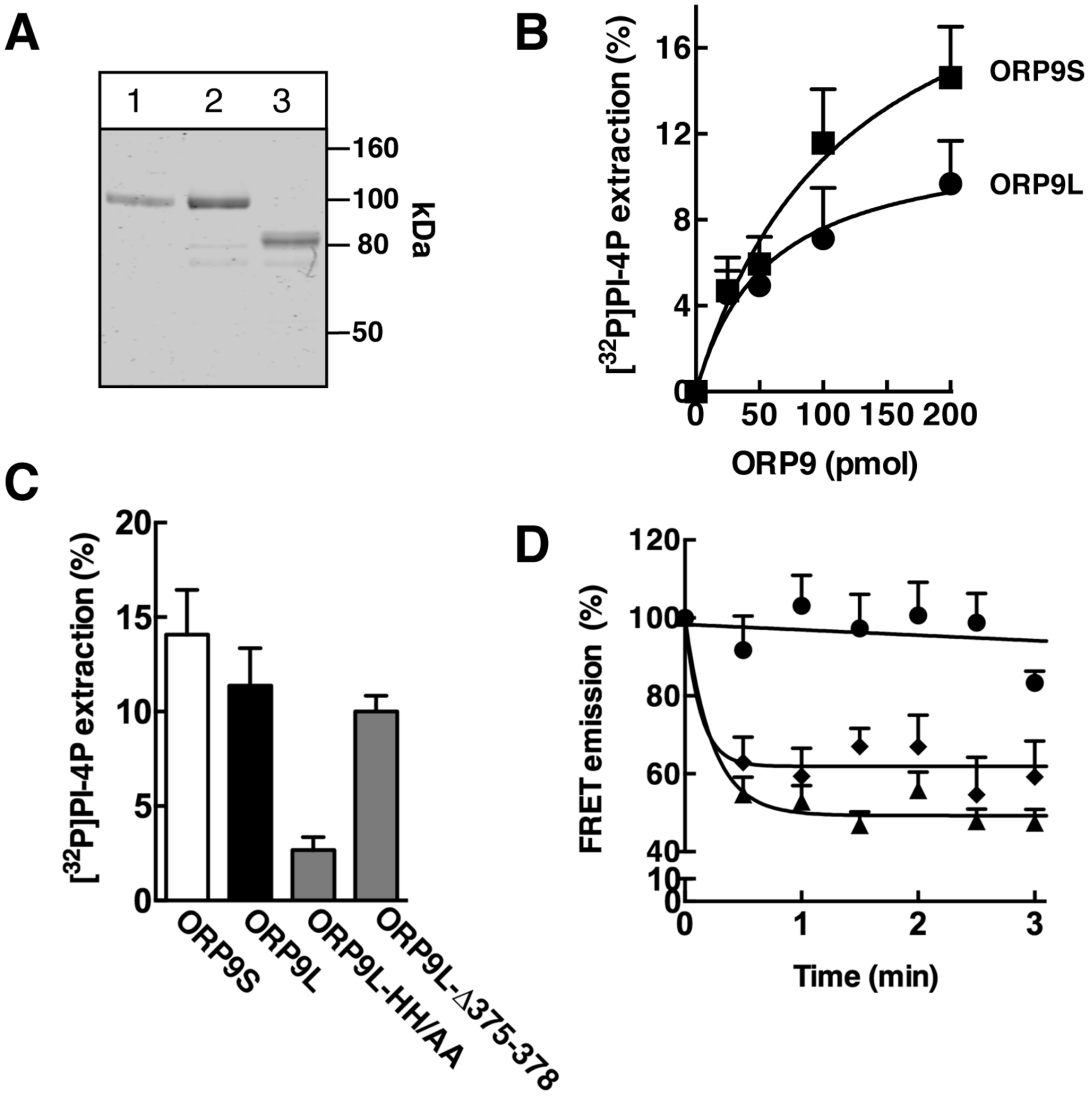
ORP9L and ORP9S are PI-4P binding proteins. A, ORP9L (lane 1), ORP9L-HH/AA (lane 2) and ORP9S (lane 3) (0.8 µg each) were purified from baculovirus-infected Sf21 cells, resolved by SDS-8% PAGE and stained with Coomassie Blue. B, the extraction of [^32^P]PI-4P from liposomes by increasing amounts of ORP9L or ORP9S was assayed at 25°C for 20 min as described in Experimental Procedures. Percent extraction is based on total [^32^P]PI-4P input in each assay and corrected for background in the absence of protein. Results are mean and SEM of 3 experiments. C, [^32^P]PI-4P extraction by ORP9S and ORP9L mutants (100 pmol) was assayed as described in panel B. Results are the mean and SEM of 3 experiments. D, CTL extraction from liposomes by no addition (l), ORP9L (⧫) or ORP9L-HH/AA (▴) (2.5 µM). Results are the mean and SEM of 3 experiments.

ORP9 has a conserved histidine pair at position 488 and 489 that could mediate binding of the inositol 4-phosphate headgroup of PI-4P. To test this, ORP9L, ORP9S and ORP9L-HH_488,489_AA (HH/AA) were purified from baculovirus-transducted Sf21 cells by metal affinity chromatography (Fig. 3A). Purified ORP9L and ORP9S were incubated with liposomes containing [^32^P]PI-4P and extraction of radioactivity into the supernatant was assayed (Fig. 3B and C). Increasing concentrations of ORP9L extracted a maximum of 9% [^32^P]PI-4P from liposomes (Fig. 3B), which was similar to OSBP extraction of PI-4P under the same conditions [18]. ORP9S extracted slightly more PI-4P (14%) indicating the PH domain does not significantly affect this activity. Mutation of the conserved histidine pair in ORP9L-HH/AA reduced PI-4P extraction relative to wild-type ORP9L and ORP9S by >75% (Fig. 3C). Mutation of conserved residues in the β-barrel lid of ORP9L-Δ375–378 that reduce affinity for cholesterol [22] did not affect PI-4P extraction activity. The sterol extraction activity of ORP9L-HH/AA was tested in the CTL-based assay (Fig. 3D). ORP9L-HH/AA extracted CTL from liposomes as efficiently as the wild-type protein confirming that these residues are involved specifically in PI-4P binding.

**Figure 4:**
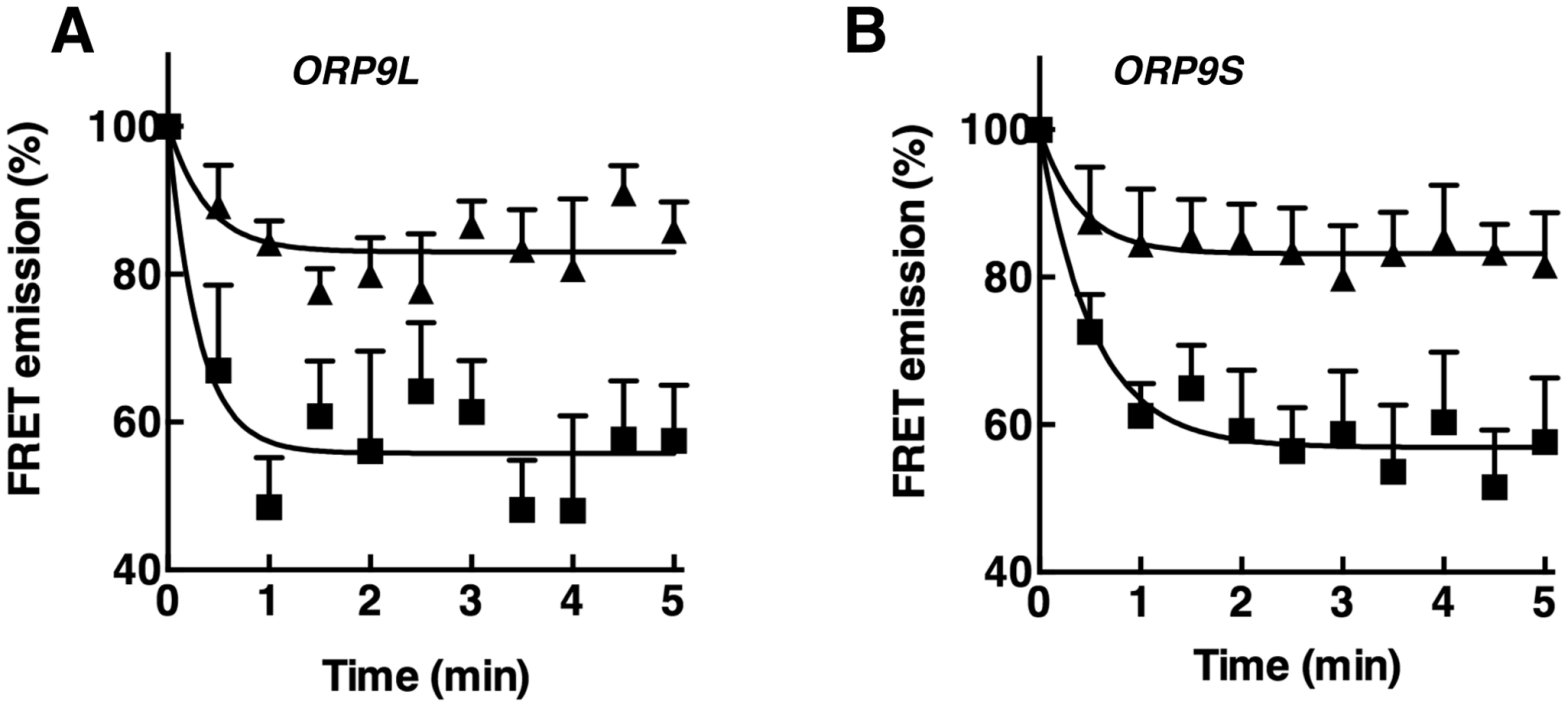
PI-4P inhibits extraction of CTL from liposomes by ORP9L and ORP9S. The extraction of CTL by ORP9L (panel A) and ORP9S (panel B) was assayed using liposomes containing 0 (▪) or 2.5 mol% (▴) PI-4P. Results are the mean and SEM of 4–9 experiments.

The ability of PI-4P to compete with sterols was measured by including PI-4P in liposomes containing CTL (2.5 mol%) and measuring extraction by ORP9L and ORP9S relative to PI-4P-free liposomes (Fig. 4). Inclusion of an equimolar amount to PI-4P (2.5 mol%) inhibited CTL extraction by both ORP9L and ORP9S (Fig. 4A and B). Increasing the PI-4P content above 2.5 mol% did not further inhibit CTL extraction by either ORP9L or ORP9S (results not shown).

**Figure 5:**
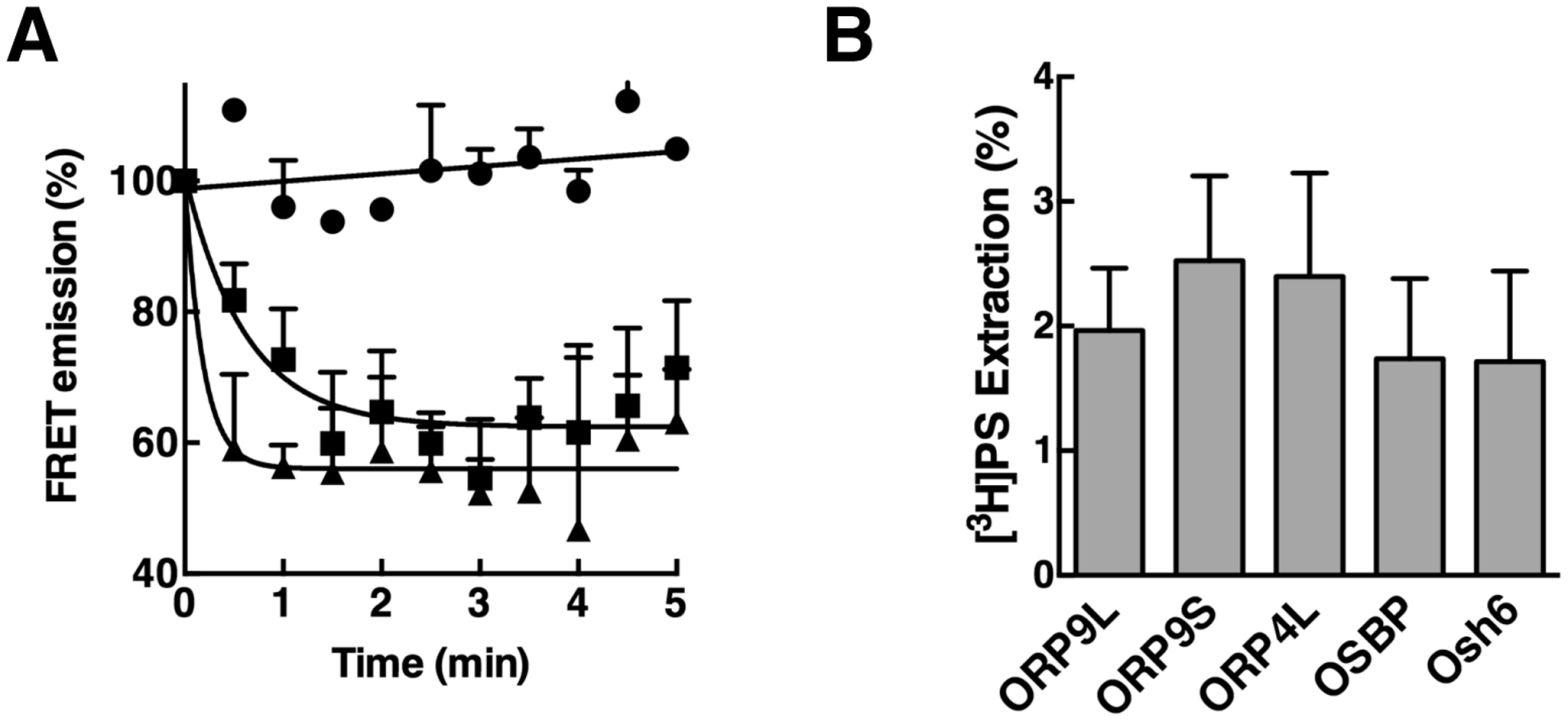
Evaluation of PS binding by ORP9L or ORP9S. A, CTL extraction by ORP9L (▪) and ORP9S (▴) was measured as described in the legend to Fig. 1E except liposomes were devoid of PS. Control assays (•) contained no protein. B, the indicated proteins (100 pmol) were incubated with liposomes containing 2.5 mol% [^3^H]serine-labelled PS and extraction of radioactivity into the supernatant was measured as described in Materials and Methods. Background PS extraction activity in the absence of protein was 3.2±2.3%. Results for both experiments are the mean and SEM of 3–6 experiments.

A recent study that identified Osh6 and Osh7 as PS binding and transfer proteins also suggested that several mammalian ORPs, including ORP9, might have similar activity based on the presence of a conserved α1-β2 loop at the entrance to the binding pocket and lid that interacts with phosphoserine [16]. Since CTL was extracted from liposomes containing 10 mol% PS, it was deemed unlikely to be a strong competitive ligand for ORP9. In support of this conclusion, we found that the amount of CTL extracted by ORP9L and ORP9S from PS-free liposomes (Fig. 5A) was similar to experiments using 10 mol% PS liposomes (see Fig. 1E). Direct extraction of [^3^H]PS from liposomes by ORP9 was also measured using an assay similar to that used to measure PI-4P binding (Fig. 5B). The PS-binding protein Osh6 extracted approximately 2% of [^3^H]PS from liposomes, which is similar to activity reported using a nonradioactive-based assay [16]. ORP9L, ORP9S, OSBP and ORP4 had similar activity, suggesting that PS is potential ligand but is relatively weak compared to sterols and PI-4P.

### Expression of ORP9 sequesters cellular PI-4P

**Figure 6:**
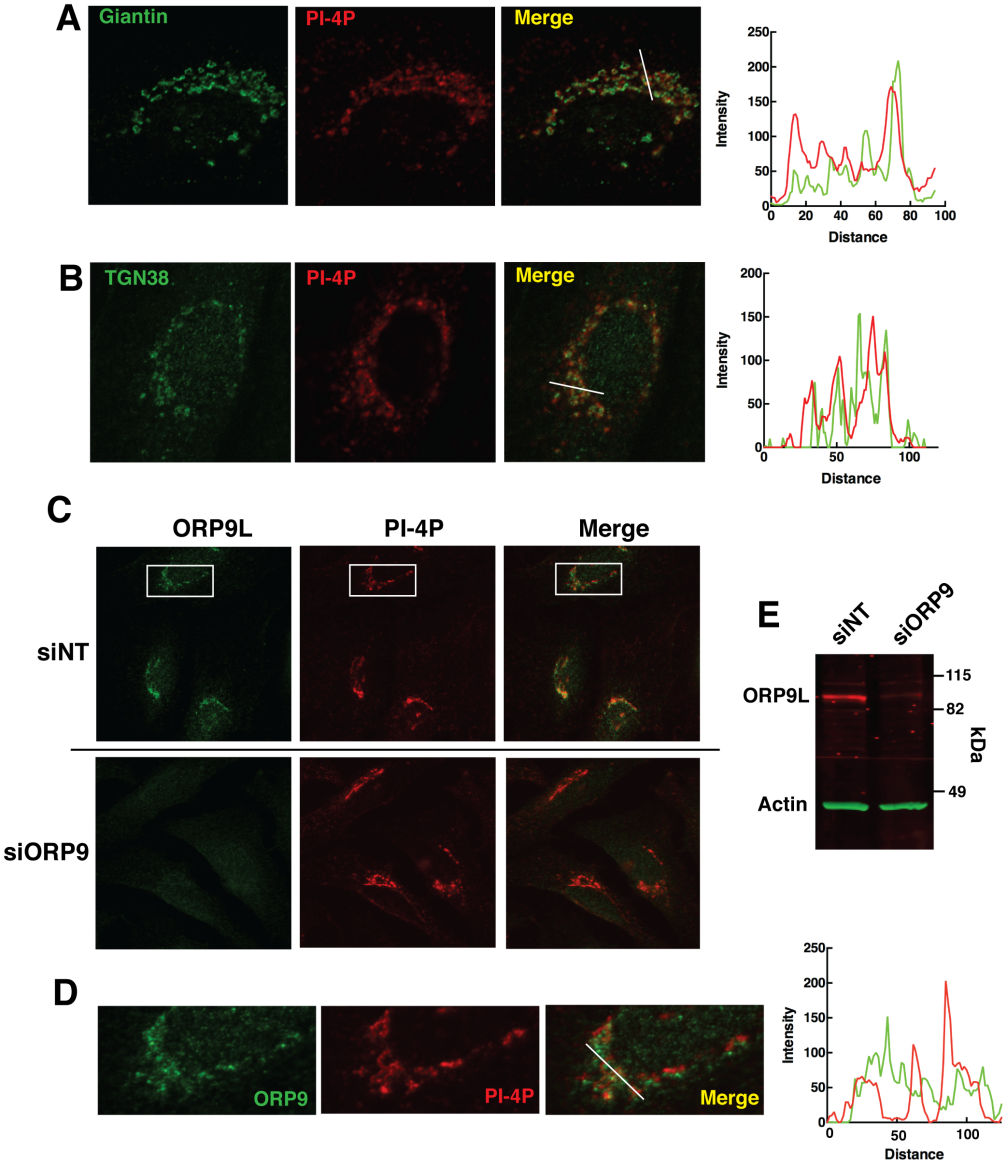
Silencing of ORP9L expression by RNAi does not affect the Golgi distribution of PI-4P. A and B, Hela cells were co-immunostained for PI-4P and giantin (Panel A) or TGN38 (Panel B). Line plots adjacent to each panel show the relative fluorescence intensity of the Golgi markers (green) and PI-4P (red) imaged along the white line shown in the merged image. C and D, ORP9L expression in HeLa cells was silenced by transfection of non-targeting (siNT) or ORP9L-specific siRNAs for 48 h. Cells were fixed and immunostained with anti-ORP9 polyclonal and PI-4P monoclonal antibodies, followed by Alexafluor488 and Alexafluor594 secondary antibodies, respectively. Epifluorescence images were captured using identical exposure times and microscope settings. Panel D shows an enlargement of the boxed regions indicated in siNT transfected HeLa cells in Panel C. The adjacent line plot shows the fluorescent intensity of ORP9L (green) and PI-4P (red) along the white line in the merged image. Panel E, lysates of siNT- and siORP9-transfected HeLa cells were immunoblotted with ORP9 and actin primary antibodies, IRDye 680- and 800-conjugated secondary antibodies, and imaged using a Licor Odyssey.

ORP9L is localized to the trans-Golgi/TGN where it affects Golgi secretion and the distribution of cholesterol in post-Golgi compartments [22]. Since PI-4P is synthesized in late Golgi compartments and has an essential role in recruitment of secretory factors [30]–[32], we tested whether reduction of ORP9L expression by siRNA silencing affected the distribution or content of PI-4P in that compartment. PI-4P was visualized in HeLa cells using a PI-4P-specific monoclonal antibody that does not recognize other phosphatidylinositol species [33]. PI-4P immunostaining was primarily in a perinuclear region that partially overlapped with the cis/medial marker giantin and the trans-Golgi network protein TGN38 (Fig. 6A and B). In HeLa cells transfected with control non-targeting siRNA (siNT), ORP9L and PI-4P were poorly co-localization in the perinuclear Golgi compartment (Fig. 6C and D). siRNA silencing of ORP9L reduced expression by >80% compared to siNT transfected HeLa cells (Fig. 6E) but the staining intensity and distribution of PI-4P was similar to control cells (Fig. 6C).

**Figure 7:**
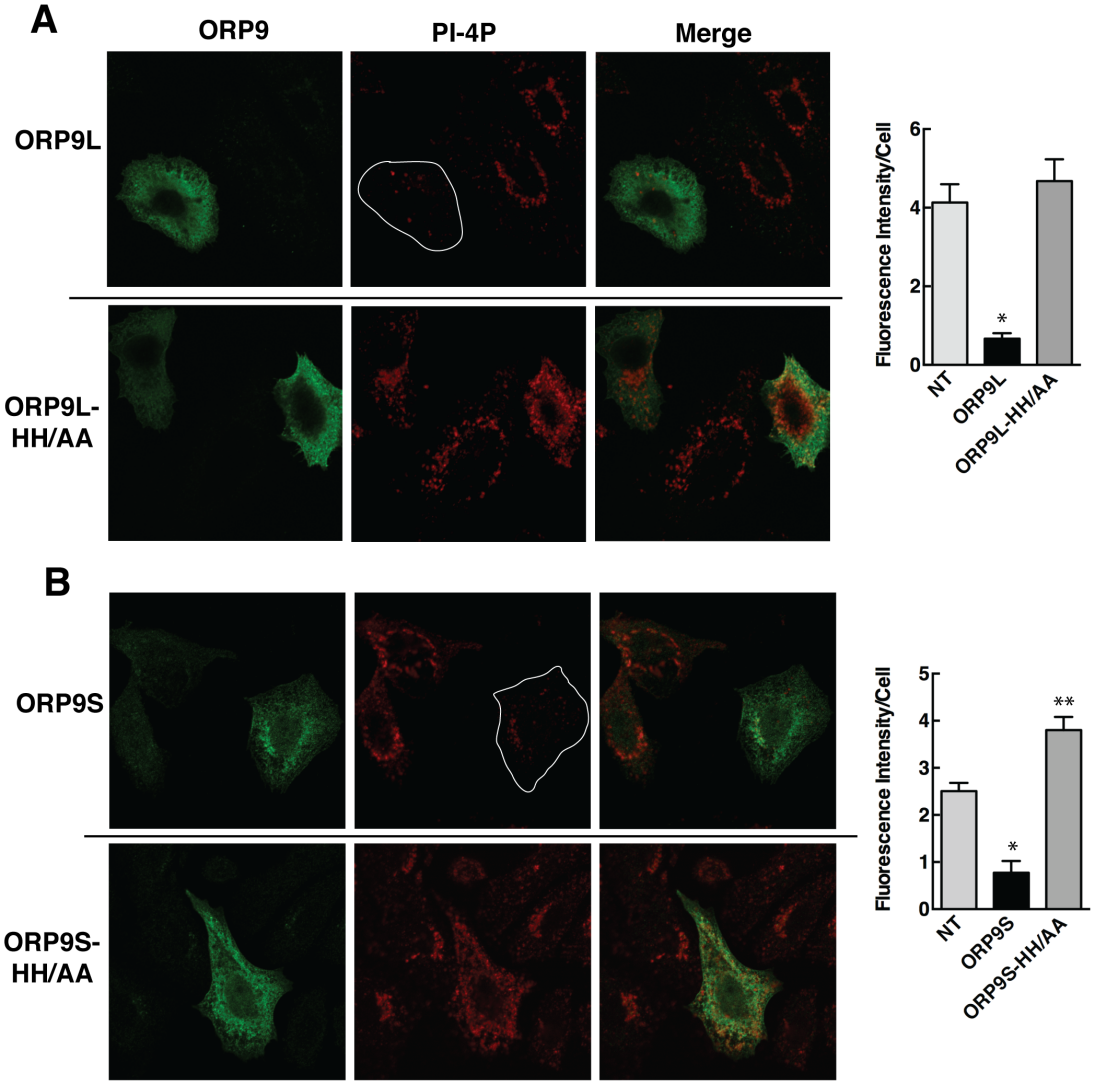
Sequestration of PI-4P by ORP9L and ORP9S in CHO cells is prevented by the HH_488,489_AA mutation. CHO cells were transiently transfected with vectors encoding the wild-type and HH/AA mutant of ORP9L (panel A) or the wild-type and the HH/AA mutant of ORP9S (panel B) for 48 h. Cells were fixed and immunostained for ORP9 and PI-4P as described in the legend to Fig. 6. Confocal images (0.8 µm scans) were captured using identical settings. The outlines of ORP9L and ORP9S expressing cells are shown in PI-4P panels. Plots adjacent to Panels A and B show the fluorescence intensity in non-transfected (NT) cells compared to those expressing wild-type or HH/AA mutants of ORPL and ORP9S. Results are the mean and SEM of 3 experiments (20–50 cells). *p<0.002 and **p<0.01 compared to NT controls.

To determine if ORP9 could sequester or alter PI-4P distribution at the Golgi apparatus, ORP9L and ORP9S, as well as the HH/AA mutants that are defective in PI-4P binding, were transiently expressed in CHO cells and the localization of PI-4P was monitored by immunofluorescence confocal microscopy (Fig. 7). CHO cells expressing ORP9L displayed an 80% reduction in PI-4P fluorescence intensity compared to surrounding non-transfected cells (Fig. 7A). ORP9L-HH/AA was diffusely localized like the wild-type protein but the distribution and intensity of PI-4P immunostaining in expressing cells was not significantly affected. ORP9S expression also reduced PI-4P immunostaining relative to non-transfected cells (Fig. 7B). Interestingly, ORP9S-HH/AA expression increased PI-4P fluorescence intensity throughout the cell. Thus excess ORP9 shields PI-4P from detection or dramatically reduces Golgi PI-4P content (Fig. 7A and B).

**Figure 8:**
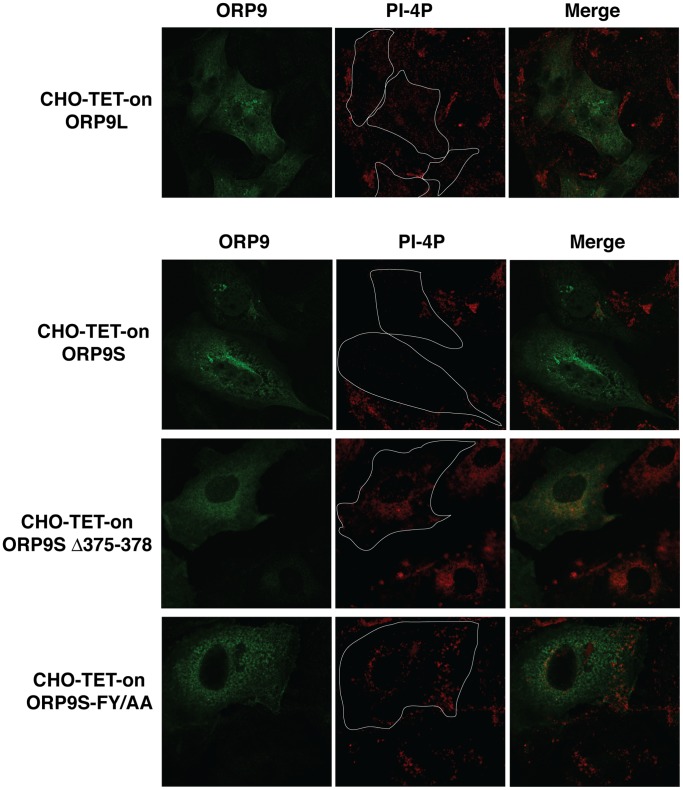
Sequestration of Golgi PI-4P by inducible overexpression of ORP9S requires VAP and sterol binding. CHO cells stably expressing ORP9L, ORP9S, ORP9S- Δ375–378 or ORP9S-FY/AA under the control of the TET-on transactivator were induced in medium containing 1 µg/ml doxycycline for 24 h. After fixing and permeabilization, ORP9L, ORP9S and PI-4P were detected with anti-ORP9 polyclonal and PI-4P monoclonal antibodies as described in the legend to Fig. 6. Confocal images (0.8 µm scans) were captured using identical settings. The outlines of ORP9L and ORP9S expressing cells are shown in PI-4P panels.

Overexpression of ORP9L and ORP9S cause disorganization of the Golgi apparatus and, in the case of ORP9S, inhibit secretion and cell proliferation [22]. This was prevented by mutations that disrupted VAP binding by the FFAT motif (ORP9-FY/AA) and sterol binding (ORP9S-Δ375–378). To assess if these effects could be related to sequestration of PI-4P, CHO cells expressing ORP9L and ORP9S under the control of the TET-on transactivator were cultured in the presence of doxycycline for 48 h and immunostained for PI-4P (Fig. 8). Similar to the results shown in Fig. 7, CHO cells expressing ORP9L or ORP9S were virtually devoid of PI-4P staining (see outlined areas in PI-4P panels) compared to adjacent non-transfected cells. In contrast, expression of ORP9S-FY/AA or ORP9S-Δ375–378 had no effect on PI-4P detection compared to adjacent non-transfected cells, indicating that the VAP and sterol binding domains of ORP9S allows it to access and interact with PI-4P in the Golgi/cytoplasmic compartment.

## Discussion

Emerging evidence of divergent ligand specificities among OSBP homologues suggests regulatory functions in sterol/anionic phospholipid homeostasis and transport. OSBP, the prototypic member originally identified by its 25OH binding activity, binds PI-4P competitively with cholesterol and may exchange these lipids or regulate associated metabolic pathways at the ER-Golgi interface. ORP9 and ORP11 also associate with the Golgi apparatus where they influence the structure of and cholesterol distribution in the ER-Golgi intermediate compartment, Golgi apparatus and late endosomes [22], [25], [34]. ORP9 is a cholesterol binding protein but could also bind PI-4P and PS via conserved residues at the entrance to its lipid-binding pocket. Similar to OSBP, results show that Golgi-localized ORP9L, as well as the ORP9S variant lacking the PH domain, are cholesterol and PI-4P binding proteins that sequester and/or modify Golgi PI-4P content when expressed in cells.

An assay using the FRET pair CTL and dansyl-PE was developed to measure the sterol extraction and transfer activity OSBP and ORP9. Based on a competition assay between soluble sterols, CTL and DHE were both poor ligands for OSBP relative to cholesterol. The lack of extraction of CTL or DHE from liposomes by OSBP thus reflects poor affinity as well as the fact that OSBP extracts only 10–15% of the preferred ligand [^3^H]cholesterol under similar conditions. A recent study showed that DHE can be efficiently extracted and transferred between liposomes but only when OSBP is activated by trypsin digestion or by interaction with VAP and PI-4P in donor and acceptor membranes [15]. In contrast, CTL extraction by ORP9L and ORP9S was rapid and reached a stable baseline within 90–120 s. Despite some variability in the activity of ORP9L and ORP9S preparations purified from Sf21 cells, perhaps reflecting the presence of an endogenous ligand or modification, a 1∶1 (mol/mol) ratio of ORP9 to liposomal CTL reproducibly yielded 30–40% extraction. Although we were unable to directly compare the affinity of CTL and cholesterol using the competition assays described in Fig. 1B, the robust activity measured in the FRET assay compared to previous measures of [^3^H]cholesterol extraction from liposomes [22] indicates that ORP9L and ORP9S could remain associated with the liposomes after extraction of CTL. It seems unlikely that ORP9 has increased affinity for CTL since the addition of equimolar cholesterol to liposomes inhibited ORP9L extraction of CTL by 50% (Fig. 4C). Despite robust extraction of CTL by ORP9L and ORP9S, we were unable to demonstrate transfer to acceptor liposomes under a variety of conditions including in the presence of PI-4P and recombinant VAPA (result not shown). The reasons for this remain unclear since [^3^H]cholesterol transfer between liposomes by ORP9L using a precipitation method was previously observed. The discrepancy could be due to differences in the sterol ligands or the type of assay. A FRET-based assay is the preferred method since it measures the delivery of sterol to the donor membrane and does not register transfer proteins that bind to membranes but do not release their ligand, which could occur in assays that rely on liposome precipitation. However, a caveat of FRET assays is the use non-physiological ligands that have reduced affinity compared to endogenous ligands, as is the case for DHE binding by OSBP.

The results of direct binding and competition assays, and mutagenesis of essential residues indicate that ORP9L and ORP9S bind PI-4P in the C-terminal lipid-binding pocket. The ORP9 PH domain, which recognizes PI-4P in the Golgi apparatus [22], does not appear to affect this activity since ORP9L and ORP9S extracted PI-4P to a similar degree. Importantly, ORP9L-HH_488,489_AA and ORP9L Δ375–378 had normal CTL and PI-4P binding activity, respectively, indicating that binding of these ligands is mutually exclusive but involves interaction with different residues in the binding pocket. CTL extraction from liposomes by ORP9 was inhibited 50% by inclusion of an equimolar amount of PI-4P indicating similar relative affinities for both ligands. A PS recognition motif identified in Osh6 and Osh7 is conserved in several ORPs including ORP9 [16], and ORP9, OSBP and ORP4 displayed similar PS extraction activity as Osh6. However, inclusion of PS in liposomes did not affect CTL extraction by ORP9L or ORP9S and the relative extraction efficiently of sterols and PI-4P by ORP9 was greater, suggesting that PS is a weaker, non-preferred ligand *in vitro*. Whether ORP9 or other members of the family affect PS metabolism or distribution in cells requires further study.

PI-4P is enriched in the trans-Golgi/TGN where binding or transport by ORP9L could regulate its distribution or content, accounting for the inhibition of ER-Golgi protein transport and altered Golgi structure observed following ORP9L silencing in CHO cells [22]. Despite the trans-Golgi/TGN localization of ORP9L there was minimal co-localization with PI-4P, suggesting that ORP9L could prevent PI-4P accumulation in its Golgi compartment or sequester PI-4P from detection by the anti-PI-4P antibody. However, silencing of ORP9L did not effect PI-4P distribution indicating minimal impact on the metabolism and partitioning of PI-4P within the Golgi apparatus. We cannot discount the possibility that loss of ORP9L expression might alter the metabolism of PI-4P without affecting immunodetection of the Golgi pool, or be compensated for by OSBP or other Golgi-specific PI-4P metabolic pathways.

Inducible expression of ORP9S inhibits ER-Golgi protein transport, fragments the Golgi apparatus and inhibits cell proliferation [22], which could be related to PI-4P sequestration. Indeed, inducible or transient expression of ORP9S or ORP9L blocked immunodetection of PI-4P, which was prevented by mutation of two histidine residues required for PI-4P binding *in vitro*. A prior study showed that immunodetection of PI-4P is competed by exogenous addition of the FAPP-PH domain to fixed cells [33], suggesting that the observed loss of PI-4P immunofluorescence is due to binding of PI-4P by overexpressed ORP9. However, we cannot rule out the possibility that ORP9 expression facilitates a reduction in the PI-4P content of Golgi membranes. Our finding that ORP9S sequestered PI-4P but the FFAT and sterol-binding mutants did not is consistent with the lack of growth inhibition by these two mutants [22]. It is feasible that VAP and sterol binding activity of ORP9S allows it to access and remove PI-4P from its essential secretory functions in the Golgi apparatus. In contrast, ORP9L also sequestered PI-4P but because ORP9L associates with the Golgi apparatus via the PH domain, PI-4P could still be available to promote secretion from the compartment. Thus the absence of a PH domain in ORP9S does not directly affect its ability to sequester Golgi PI-4P but could limit the subsequent availability of PI-4P.

In conclusion, we have identified another member of the mammalian OSBP family that binds sterols and PI-4P at the ER-Golgi interface. Although ORP9L and OSBP are situated in the trans-Golgi/TGN, there are fundamental differences in terms of lipid binding properties and protein partners that reveal different functional outputs. Whether there is a common underlying transport or signalling activity remains to be resolved.

## References

[1] Olkkonen VM, Li S (2013) Oxysterol-binding proteins: sterol and phosphoinositide sensors coordinating transport, signaling and metabolism. Prog Lipid Res 52: 529–538. 23830809 10.1016/j.plipres.2013.06.004

[2] Ngo MH, Colbourne TR, Ridgway ND (2010) Functional implications of sterol transport by the oxysterol-binding protein gene family. Biochem J 429: 13–24. 20545625 10.1042/BJ20100263

[3] Im YJ, Raychaudhuri S, Prinz WA, Hurley JH (2005) Structural mechanism for sterol sensing and transport by OSBP-related proteins. Nature 437: 154–158. 16136145 10.1038/nature03923 PMC1431608

[4] Tong J, Yang H, Eom SH, Im YJ (2013) Structure of Osh3 reveals a conserved mode of phosphoinositide binding in oxysterol-binding proteins. Structure 21: 1203–1213. 23791945 10.1016/j.str.2013.05.007

[5] Wyles JP, McMaster CR, Ridgway ND (2002) Vesicle-associated Membrane Protein-associated Protein-A (VAP-A) Interacts with the Oxysterol-binding Protein to Modify Export from the Endoplasmic Reticulum. J Biol Chem 277: 29908–29918. 12023275 10.1074/jbc.M201191200

[6] Loewen CJ, Roy A, Levine TP (2003) A conserved ER targeting motif in three families of lipid binding proteins and in Opi1p binds VAP. Embo J 22: 2025–2035. 12727870 10.1093/emboj/cdg201 PMC156073

[7] Levine TP, Munro S (2002) Targeting of Golgi-specific pleckstrin homology domains involves both PtdIns 4-kinase-dependent and -independent components. Curr Biol 12: 695–704. 12007412 10.1016/s0960-9822(02)00779-0

[8] Lehto M, Hynynen R, Karjalainen K, Kuismanen E, Hyvarinen K, et al (2005) Targeting of OSBP-related protein 3 (ORP3) to endoplasmic reticulum and plasma membrane is controlled by multiple determinants. Exp Cell Res 310: 445–462. 16143324 10.1016/j.yexcr.2005.08.003

[9] Beh CT, Alfaro G, Duamel G, Sullivan DP, Kersting MC, et al (2009) Yeast oxysterol-binding proteins: sterol transporters or regulators of cell polarization? Mol Cell Biochem 326: 9–13. 19125315 10.1007/s11010-008-9999-7

[10] Suchanek M, Hynynen R, Wohlfahrt G, Lehto M, Johansson M, et al (2007) The mammalian oxysterol-binding protein-related proteins (ORPs) bind 25-hydroxycholesterol in an evolutionarily conserved pocket. Biochem J 405: 473–480. 17428193 10.1042/BJ20070176 PMC2267293

[11] Schulz TA, Choi MG, Raychaudhuri S, Mears JA, Ghirlando R, et al (2009) Lipid-regulated sterol transfer between closely apposed membranes by oxysterol-binding protein homologues. J Cell Biol 187: 889–903. 20008566 10.1083/jcb.200905007 PMC2806323

[12] Kandutsch AA, Shown EP (1981) Assay of oxysterol-binding protein in a mouse fibroblast, cell-free system. Dissociation constant and other properties of the system. J Biol Chem 256: 13068–13073. 7309751

[13] Ridgway ND, Dawson PA, Ho YK, Brown MS, Goldstein JL (1992) Translocation of oxysterol binding protein to Golgi apparatus triggered by ligand binding. J Cell Biol 116: 307–319. 1730758 10.1083/jcb.116.2.307 PMC2289278

[14] de Saint-Jean M, Delfosse V, Douguet D, Chicanne G, Payrastre B, et al (2011) Osh4p exchanges sterols for phosphatidylinositol 4-phosphate between lipid bilayers. J Cell Biol 195: 965–978. 22162133 10.1083/jcb.201104062 PMC3241724

[15] Mesmin B, Bigay J, Moser von Filseck J, Lacas-Gervais S, Drin G, et al (2013) A four-step cycle driven by PI(4)P hydrolysis directs sterol/PI(4)P exchange by the ER-Golgi tether OSBP. Cell 155: 830–843. 24209621 10.1016/j.cell.2013.09.056

[16] Maeda K, Anand K, Chiapparino A, Kumar A, Poletto M, et al (2013) Interactome map uncovers phosphatidylserine transport by oxysterol-binding proteins. Nature 501: 257–261. 23934110 10.1038/nature12430

[17] Wang C, JeBailey L, Ridgway ND (2002) Oxysterol-binding-protein (OSBP)-related protein 4 binds 25- hydroxycholesterol and interacts with vimentin intermediate filaments. Biochem J 361: 461–472. 11802775 10.1042/0264-6021:3610461 PMC1222328

[18] Goto A, Liu X, Robinson CA, Ridgway ND (2012) Multisite phosphorylation of oxysterol-binding protein regulates sterol binding and activation of sphingomyelin synthesis. Mol Biol Cell 23: 3624–3635. 22875984 10.1091/mbc.E12-04-0283 PMC3442410

[19] Perry RJ, Ridgway ND (2006) Oxysterol-binding Protein and Vesicle-associated Membrane Protein-associated Protein Are Required for Sterol-dependent Activation of the Ceramide Transport Protein. Mol Biol Cell 17: 2604–2616. 16571669 10.1091/mbc.E06-01-0060 PMC1474796

[20] Banerji S, Ngo M, Lane CF, Robinson CA, Minogue S, et al (2010) Oxysterol Binding Protein (OSBP)-Dependent Activation of Sphingomyelin Synthesis in the Golgi Apparatus Requires PtdIns 4-Kinase IIα. Mol Biol Cell. 10.1091/mbc.E10-05-0424 PMC2993743 20881054

[21] Nishimura T, Uchida Y, Yachi R, Kudlyk T, Lupashin V, et al (2013) Oxysterol-binding protein (OSBP) is required for the perinuclear localization of intra-Golgi v-SNAREs. Mol Biol Cell 24: 3534–3544. 24048449 10.1091/mbc.E13-05-0250 PMC3826991

[22] Ngo M, Ridgway ND (2009) Oxysterol binding protein-related Protein 9 (ORP9) is a cholesterol transfer protein that regulates Golgi structure and function. Mol Biol Cell 20: 1388–1399. 19129476 10.1091/mbc.E08-09-0905 PMC2649274

[23] Lessmann E, Ngo M, Leitges M, Minguet S, Ridgway ND, et al (2007) Oxysterol-binding protein-related protein (ORP) 9 is a PDK-2 substrate and regulates Akt phosphorylation. Cell Signal 19: 384–392. 16962287 10.1016/j.cellsig.2006.07.009

[24] Ohvo-Rekila H, Akerlund B, Slotte JP (2000) Cyclodextrin-catalyzed extraction of fluorescent sterols from monolayer membranes and small unilamellar vesicles. Chem Phys Lipids 105: 167–178. 10823464 10.1016/s0009-3084(00)00122-5

[25] Wyles JP, Ridgway ND (2004) VAMP-associated protein-A regulates partitioning of oxysterol-binding protein-related protein-9 between the endoplasmic reticulum and Golgi apparatus. Exp Cell Res 297: 533–547. 15212954 10.1016/j.yexcr.2004.03.052

[26] Peterson GL (1977) A simplification of the protein assay method of Lowry et al. which is more generally applicable. Analytical Biochem 83: 346–356. 10.1016/0003-2697(77)90043-4 603028

[27] Scheidt HA, Muller P, Herrmann A, Huster D (2003) The potential of fluorescent and spin-labeled steroid analogs to mimic natural cholesterol. J Biol Chem 278: 45563–45569. 12947110 10.1074/jbc.M303567200

[28] Xu Z, Farver W, Kodukula S, Storch J (2008) Regulation of sterol transport between membranes and NPC2. Biochemistry 47: 11134–11143. 18823126 10.1021/bi801328u PMC4355403

[29] Du X, Kumar J, Ferguson C, Schulz TA, Ong YS, et al (2011) A role for oxysterol-binding protein-related protein 5 in endosomal cholesterol trafficking. J Cell Biol 192: 121–135. 21220512 10.1083/jcb.201004142 PMC3019559

[30] Hausser A, Storz P, Martens S, Link G, Toker A, et al (2005) Protein kinase D regulates vesicular transport by phosphorylating and activating phosphatidylinositol-4 kinase III[beta] at the Golgi complex. Nat Cell Biol 7: 880–886. 16100512 10.1038/ncb1289 PMC1458033

[31] Wang YJ, Wang J, Sun HQ, Martinez M, Sun YX, et al (2003) Phosphatidylinositol 4 phosphate regulates targeting of clathrin adaptor AP-1 complexes to the Golgi. Cell 114: 299–310. 12914695 10.1016/s0092-8674(03)00603-2

[32] Godi A, Di Campli A, Konstantakopoulos A, Di Tullio G, Alessi DR, et al (2004) FAPPs control Golgi-to-cell-surface membrane traffic by binding to ARF and PtdIns(4)P. Nat Cell Biol 6: 393–404. 15107860 10.1038/ncb1119

[33] Hammond GR, Schiavo G, Irvine RF (2009) Immunocytochemical techniques reveal multiple, distinct cellular pools of PtdIns4P and PtdIns(4,5)P(2). Biochem J 422: 23–35. 19508231 10.1042/BJ20090428 PMC2722159

[34] Zhou Y, Li S, Mayranpaa MI, Zhong W, Back N, et al (2010) OSBP-related protein 11 (ORP11) dimerizes with ORP9 and localizes at the Golgi-late endosome interface. Exp Cell Res 316: 3304–3316. 20599956 10.1016/j.yexcr.2010.06.008

